# Off-Center Rotation of CuPc Molecular Rotor on a Bi(111) Surface and the Chiral Feature

**DOI:** 10.3390/molecules22050740

**Published:** 2017-05-04

**Authors:** Kai Sun, Min-Long Tao, Yu-Bing Tu, Jun-Zhong Wang

**Affiliations:** School of Physical Science and Technology, MOE Key Laboratory on Luminescence and Real-Time Analysis, Southwest University, Chongqing 400715, China; sun.andkai@163.com (K.S.); taotaole@swu.edu.cn (M.-L.T.); tuyubing@gmail.com (Y.-B.T.)

**Keywords:** scanning tunneling microscopy (STM), CuPc molecules, off-center rotation, semi-metallic Bi, chiral feature

## Abstract

Molecular rotors with an off-center axis and the chiral feature of achiral CuPc molecules on a semi-metallic Bi(111) surface have been investigated by means of a scanning tunneling microscopy (STM) at liquid nitrogen (LN_2_) temperature. The rotation axis of each CuPc molecular rotor is located at the end of a phthalocyanine group. As molecular coverage increases, the CuPc molecules are self-assembled into various nanoclusters and finally into two-dimensional (2D) domains, in which each CuPc molecule exhibits an apparent chiral feature. Such chiral features of the CuPc molecules can be attributed to the combined effect of asymmetric charge transfer between the CuPc and Bi(111) substrate, and the intermolecular van der Waals interactions.

## 1. Introduction

**Figure 1:**
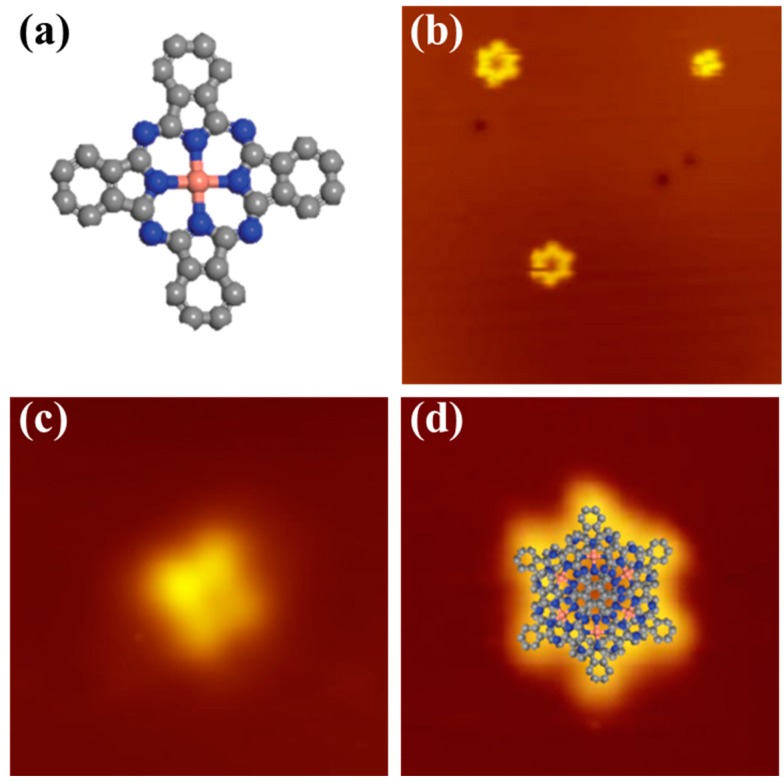
(**a**) Schematic structure model of the CuPc molecule; (**b**) STM image of isolated CuPc molecules in stationary and rotational state at 78 K, 25 × 25 nm, −2.0 V, 28 pA; (**c**) High-resolution STM image of a stationary CuPc molecule, 5 × 5 nm, −1.0 V, 26 pA; (**d**) High-resolution STM image of a rotating CuPc molecule, 5 × 5 nm, −2.8 V, 28 pA. The structural model for the six stable orientations of CuPc molecules are superposed on the STM image.

As an archetype molecule with organic semiconducting properties in the bulk, transition-metal phthalocyanines (TMPc) have attracted considerable interest due to their potential applications in organic electronic devices in the past few years [1,2,3]. Because of their high thermal stability, electronic structure, and symmetric cross-like geometrical structure in Figure 1a, TMPc can be used in organic solar cells [4], organic light emitting diodes [5], and organic field-effect transistors [6]. The interactions between TMPc and different solid surfaces have been intensively investigated by many techniques. Scanning tunneling microscopy (STM) has been proven a powerful technique for studying the geometric conformations of TMPc molecules on solid surfaces owning to the capability of high spatial resolution [7,8,9,10]. In particular, the chirality of self-assembled structures of the achiral TMPc molecules deposited on solid surfaces has been a hot topic in recent years [11,12,13,14,15,16]. Highly symmetric molecules adsorbed on a substrate with dissimilar point-group symmetry have also been shown to develop chirality through asymmetric intermolecular interactions [14,15].

So far, significant progress has been achieved in the adsorption and self-assembly of various TMPc on either noble metal surfaces such as Au [17,18,19,20,21,22,23], Ag [16,24,25,26], Cu [27,28,29,30,31,32], Pb [19,33,34,35], or inert insulating surfaces such as NaCl [36,37,38]. In the former case, the large electron density of state (DOS) at the Fermi level leads to the molecule–substrate interactions which can modify the electronic structure and magnetic properties of TMPc. In the latter case, it is possible for TMPc molecules to be charged by the tunneling electrons from STM tip such that intrinsic spin state of TMPc would be altered. An intermediate case is between the two extreme regimes such as the semi-metallic Bi(111) surface, where molecule–substrate interactions are weaker than those for metallic substrates while the charging events of TMPc molecules would be avoided. It was reported recently that pentacene grown on the semi-metallic Bi(111) substrate forms epitaxial crystalline films with the pentacene molecule standing-up even in the first monolayer [39] rather than the planar orientation on Au(111) [40], indicating the weaker molecule–substrate interactions. In addition, the isolated molecule magnet Mn_12_ without any damage was successfully grafted onto the Bi(111) surface by the tip-depositing method [41]. However, so far, investigations on the structural evolution of TMPc on semi-metallic surface from isolated TMPc molecules to full monolayer, and finally to multilayer regime, remain too rare.

In this paper, we present the off-center rotation of a single CuPc molecule on the Bi(111) surface at LN_2_ temperature, indicating the weak molecule-semimetal interaction. In the 2D self-assembled domains, the achiral CuPc molecules reveal a chiral feature such that the four phthalocyanines lobes become bent. The chiral feature of CuPc molecules can be attributed to the combined effect of symmetric charge transfer between CuPc molecules and Bi(111) substrate and the intermolecular van der Waals (vdW) interactions. Furthermore, the orientation change—from lying flat in sub-monolayer regime to standing up in multilayer regime—occurred due to the enhanced intermolecular interactions compared with the molecule–substrate interactions.

## 2. Results and Discussion

Firstly, a small amount of CuPc molecules about 0.03 monolayer (ML) were deposited onto the Bi(111) surface at room temperature (RT). Isolated CuPc molecules in stationary or rotational state were observed from the STM image, as shown in Figure 1b. The stationary molecule appears as a cross shape with four perpendicular lobes and a dark hole located at the molecular center (the Cu ion site), consistent with the structure of CuPc molecule shown in Figure 1a. The dark hole of the CuPc center can be attributed to the occupied d-orbital character away from the Fermi energy of the copper ion. This differs from the previous reports about MnPc [42] on Bi(111) that the center of molecule appears as a bright protrusion. Figure 1c is a typical topographic image of an immobile CuPc molecule on the Bi(111) surface. It is noticed that the four lobes reveal different heights, implying a tilted adsorption with one lobe touching the substrate, like the rubrene molecule adsorbed on Bi(111) [43]. We speculate that the reason why this molecule remains immobile is that it is pinned by a substrate defect.

In the absence of defect, the individual CuPc molecules keep rotating around the touching site, which serve as the off-center rotational axis. Figure 1d presents a disc-like CuPc molecule, which differs from the inherent cross-like molecular structure in Figure 1a, indicating that the individual CuPc molecule keeps off-center rotating on the Bi(111) surface. As a result, the four lobes of the CuPc molecule cannot be resolved from the STM image. The ring structure clearly exhibits a six-fold symmetry and the diameter of the rotating molecule is about 2.8 nm, which is too large for a single immobile CuPc molecule. The insert in Figure 1d shows the structural model of the rotating molecule. The observed molecular rotations indicate a weak molecule–substrate interaction between CuPc and Bi(111), whereas on noble metal substrates the molecular rotation is absent due to the considerable molecule–substrate coupling.

**Figure 2:**
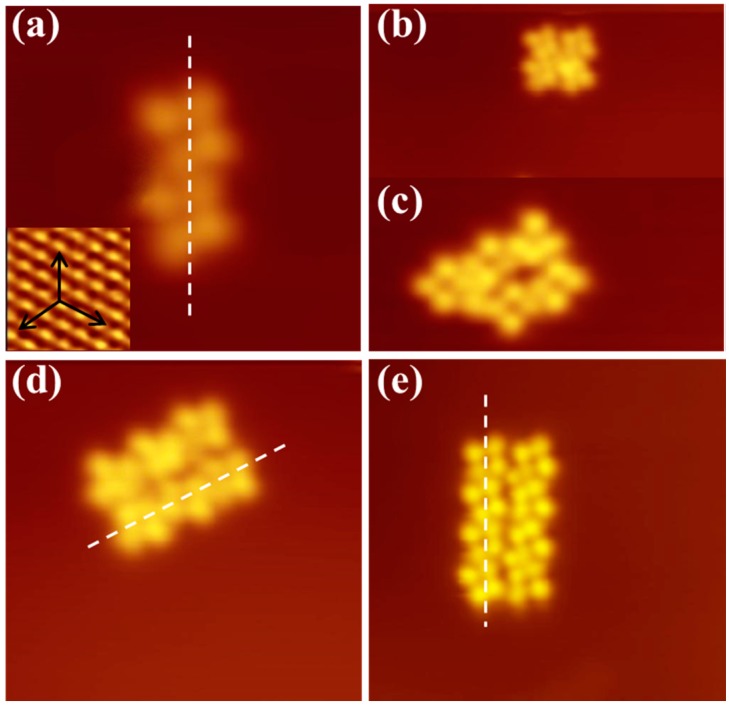
(**a**) A CuPc dimer adsorbed on the Bi(111) surface without any rotation, the arrows and dashed line represent the directions of Bi(111) surface base vectors, 6 × 6 nm, −1.7 V, 30 pA; (**b**,**c**) are the STM images of CuPc tetramer and pentamer, respectively; (**d**) STM image of a CuPc hexamer consists of two parallel molecular chains along one of the high symmetry directions of the substrate, 10 × 10 nm, −1.7 V, 36 pA; (**e**) STM image of a CuPc octamer, 12 × 12 nm, −2.0 V, 30 pA.

With molecular coverage increasing, isolated CuPc molecules began to assemble together on the Bi(111) surface, forming a series of nanoclusters—such as dimer, tetramer, hexamer, molecular chain, etc. Figure 2a shows a CuPc dimer with each monomer appearing as cross shapes consistent with its chemical structure in Figure 1a. It is noticed that the molecular rotation was absent when individual CuPc molecules assembled into clusters because of the additional van der Waals interactions between CuPc molecules. Figure 2b,c presents a tetramer and pentamer, respectively. Figure 2d,e exhibits the CuPc hexamer and octamer consisting of two parallel molecular chains. Furthermore, it is observed from the high-resolution STM images that the two opposing lobes of each CuPc molecule still remain in the directions of principal axis of the Bi(111) substrate. The alignment of the CoPc chains is also parallel to the principal axes of the Bi(111) lattice, similar to the CoPc chain adsorbed on Bi(111) [44]. It indicates that the molecule–substrate interaction is not negligible, which may arise from the surface states of the Bi(111) substrate.

**Figure 3:**
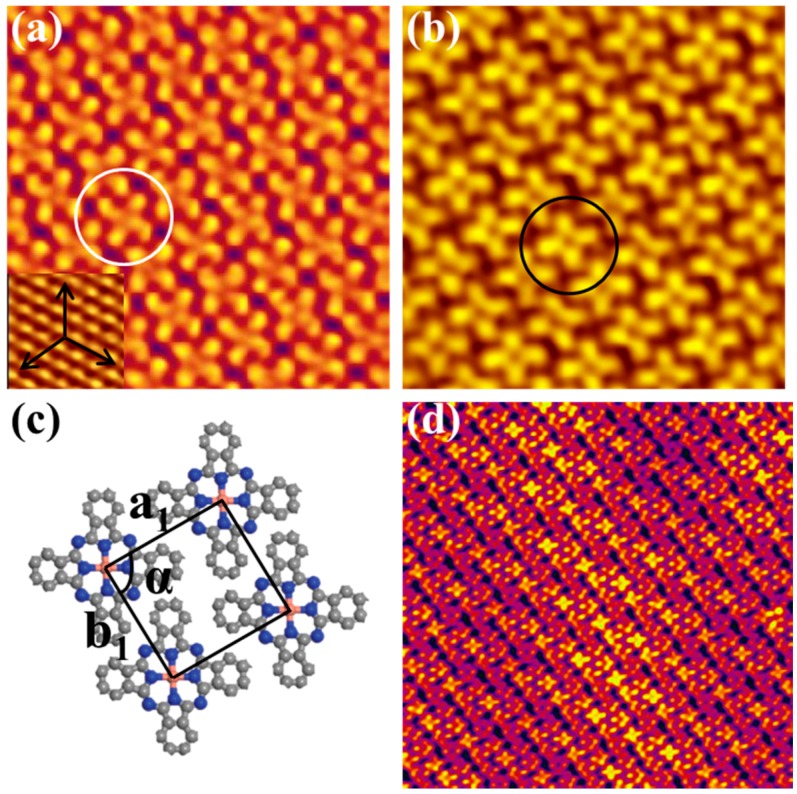
(**a**) Chiral feature appeared in the filled state STM image of the two-dimensional domains, 15 × 15 nm, −1.4 V, 28 pA; (**b**) Chiral feature disappeared essentially in the empty state STM image of the 2D domains, 10 × 10 nm, +0.5 V, 28 pA; (**c**) The structural model of the flat-lying 2D domain; (**d**) Morié fringe observed in the 2D domain of CuPc, 30 × 30 nm, −2.0 V, 28 pA.

When further depositing CuPc molecules on the Bi(111) surface, the individual molecular chains aggregated into 2D domains with parallel arrangement. Figure 3a,b shows the STM images of CuPc assembled films in the coverage of 0.75 ML acquired at −1.4 V and +0.5 V, respectively. It is observed that each CuPc molecule of the 2D domain exhibits a cross shape with four perpendicular lobes, consistent with the four-fold symmetry of CuPc molecular structure. This means that the CuPc molecules adopt a flat-lying adsorption orientation. Figure 3c displays the structural model of the 2D domains, the lattice constants of the unit cell are a_1_ = (1.42 ± 0.02) nm, b_1_ = (1.35 ± 0.02) nm, and a_1_ is aligned at one of the three principal axes of the Bi(111) surface. The angle α between a_1_ and b_1_ is measured to be 89° ± 2° corresponding with a packing density of 0.52 nm^−2^, which is smaller than that of CoPc 2D domain on the Bi(111) substrate. Unlike MnPc and CoPc, 2D domains consisting of two different molecular orientations on the Bi(111) surface, the self-assembled CuPc 2D domains have identical in-plane orientations due to the mutual coupling of the symmetry of Bi(111) and CuPc molecules. The apparent Morié fringes are also observed from the STM image in Figure 3d, which shows the epitaxial growth for CuPc molecules on the semimetal bismuth substrate.

More interestingly, the CuPc molecules of the 2D domain reveal different features under positive and negative bias voltage. Figure 3a is a STM image acquired at −1.4 V that all the CuPc molecules exhibit a strong chiral feature, especially for the CuPc molecule marked by the while circle. The two opposing lobes of each CuPc molecule are twisted toward opposite directions, revealing the asymmetric intermolecular vdW interactions. However, the chiral feature disappears in the STM image in Figure 3b obtained at +0.5 V. This phenomenon is similar to CuPc/Ag (100) system [24], but different from the MnPc and CoPc molecules. The latter reveals no chirality at both positive and negative bias voltage in 2D domains on the Bi(111) surface [42,44]. The voltage-dependent chiral appearance indicates that the original effect of molecular chirality is not a molecular geometric effect, but an electronic effect. The formation mechanism can be attributed to the combined effect of asymmetric charge transfer between CuPc molecules and Bi(111) substrate and the asymmetric intermolecular vdW interactions.

**Figure 4:**
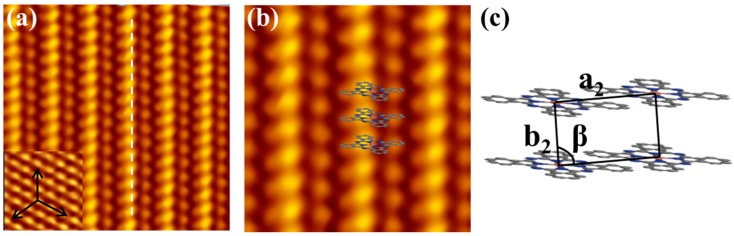
(**a**) High resolution STM image of the second layer of CuPc films consist of parallel standing-up CuPc chains, 7nm × 7 nm, −0.8 V, 28 pA; (**b**) Zoomed-in STM image of the second layer domain, in which the CuPc molecules are marked by the superposed structural models; (**c**) The structural models of the unit cell in domain (**a**).

In order to investigate the structural evolution of CuPc molecules in the multilayer regime, more CuPc molecules were deposited onto the substrate at RT subsequently. When the molecular coverage exceeds 1 ML, the second CuPc layers were achieved on the surface. Figure 4a is the high-resolution STM image of the second layer, in which each CuPc molecule adopts the standing-up adsorption orientation, rather than the planar orientation of the underlying layer. Figure 4b is the zoomed-in STM image of the second CuPc layer, it can be observed that the pure domain is composed of several parallel molecular chains, in which each CuPc molecules adopts the face to face alignment. The distance of the CuPc molecules in the same chain is smaller than that in the neighboring chains, indicating that the intermolecular interactions for the former case are obviously stronger than the latter case. The structural model of unit cell of the second layer thin film is displayed in Figure 4c. The lattice constant are a_2_ = (1.18 ± 0.02) nm, b_2_ = (0.45 ± 0.02) nm, and a_2_ is parallel to one of the three principal axes of the Bi(111) surface. The angle β between a_2_ and b_2_ is measured to be 85° ± 2° and the packing density is calculated to be 1.87 nm^−2^, which is 27.8% larger than that of the flat-lying CuPc layer. More importantly, an orientational transition from lying flat in the first layer to standing up in the second layer occurs due to the enhanced intermolecular interactions which can dominant the molecule–substrate interactions. Similar behavior was observed for SnPc adsorption on NaCl, intermolecular interactions dominate over the molecule-NaCl coupling and result in a tilted adsorption configuration [45].

## 3. Experiment Section

The experiments were carried out in a Unisoku low-temperature STM system with base pressure 1.0 × 10^−10^ Torr. The flat and well-ordered Bi(111) film was prepared by depositing 20 monolayers of bismuth atoms on a Si(III) 7 × 7 surface at RT with subsequent annealing at 400 K for nearly 2.5 h [46]. After overnight degassing, CuPc molecules (Sigma-Aldrich, St. Louis, MO, USA. 99% purity) were thermally sublimated from a homemade Ta boat heated to about 480 K and then were deposited onto Bi(111) film at a rate of 0.45 ML (here we define 1 ML as the amount of deposited CuPc molecules cover entirely the substrate surface) per minute while the substrate was kept at RT. A constant current mode and polycrystalline tungsten tips after e-beam heating in the molecular beam epitaxy (MBE) chamber were used for STM imaging. All the STM images were obtained at liquid nitrogen temperature (78 K).

## 4. Conclusions

In summary, we have observed the molecular rotors of CuPc molecules with an off-center axis on a semi-metallic Bi(111) surface. The rotation axis is located at the end of a phthalocyanine group. The off-center rotation of CuPc molecules can be attributed to the tilted adsorption of CuPc molecule on Bi(111) surface. Furthermore, the chiral feature has also been found in the filled state STM image of the achiral CuPc molecules, which demonstrates that the chirality arising from the combined effect of asymmetric charge transfer between CuPc molecules and Bi(111) substrate and the asymmetric intermolecular vdW interactions.
